# Development of Novel Micro-dystrophins with Enhanced Functionality

**DOI:** 10.1016/j.ymthe.2019.01.002

**Published:** 2019-02-01

**Authors:** Julian N. Ramos, Katrin Hollinger, Niclas E. Bengtsson, James M. Allen, Stephen D. Hauschka, Jeffrey S. Chamberlain

**Affiliations:** 1 Molecular and Cellular Biology Program, University of Washington School of Medicine, Seattle, WA 98195, USA; 2 Department of Neurology, University of Washington School of Medicine, Seattle, WA 98195, USA; 3 Senator Paul D. Wellstone Muscular Dystrophy Specialized Research Center, Seattle, WA 98195, USA; 4 Department of Biochemistry, University of Washington School of Medicine, Seattle, WA 98195, USA; 5 Department of Medicine, University of Washington School of Medicine, Seattle, WA 98195, USA

**Keywords:** Duchenne muscular dystrophy, DMD, dystrophin, gene therapy, AAV, clinical trials

## Abstract

Gene therapies using adeno-associated viral (AAV) vectors have advanced into clinical trials for several diseases, including Duchenne muscular dystrophy (DMD). A limitation of AAV is the carrying capacity (∼5 kb) available for genes and regulatory cassettes (RCs). These size constraints are problematic for the 2.2-Mb dystrophin gene. We previously designed a variety of miniaturized micro-dystrophins (μDys) that displayed significant, albeit incomplete, function in striated muscles. To develop μDys proteins with improved performance, we explored structural modifications of the dystrophin central rod domain. Eight μDys variants were studied that carried unique combinations of between four and six of the 24 spectrin-like repeats present in the full-length protein, as well as various hinge domains. Expression of μDys was regulated by a strong but compact muscle-restricted RC (CK8e) or by the ubiquitously active cytomegalovirus (CMV) RC. Vectors were evaluated by intramuscular injection and systemic delivery to dystrophic *mdx*^4cv^ mice, followed by analysis of skeletal muscle pathophysiology. Two μDys designs were identified that led to increased force generation compared with previous μDys while also localizing neuronal nitric oxide synthase to the sarcolemma. An AAV vector expressing the smaller of these (μDys5) from the CK8e RC is currently being evaluated in a DMD clinical trial.

## Teaser

Gene therapy for DMD is complicated by the enormous dystrophin gene. AAV vectors can only deliver partially functional micro-dystrophin genes. In this report, improved micro-dystrophin cassettes are described that increase muscle strength, avoid muscle-tendon injury, and persist more than 2 years. These improved vectors have great promise for DMD therapy.

## Introduction

Duchenne muscular dystrophy (DMD) is a recessively inherited muscle wasting disorder afflicting approximately 1 in 5,000 newborn males.^1^ Patients carry a mutation in the dystrophin (*DMD*) gene, resulting in aberrant or absent expression of the dystrophin protein. Affected individuals experience progressive wasting of skeletal muscles and cardiac dysfunction, leading to the loss of ambulation and premature death, primarily due to cardiac or respiratory failure. Current treatments are only able to slow progression of the disorder.^1^ Gene therapy approaches for DMD have been effectively applied in dystrophic animal models by either directly targeting a class of mutations, as with exon skipping or gene editing,^2^, ^3^, ^4^, ^5^ or by delivering a synthetic version of the dystrophin or utrophin gene.^6^ Vectors derived from several serotypes of adeno-associated virus (AAV) are promising vehicles for gene therapy, as they enable systemic gene delivery to muscles and have been tested extensively in clinical trials.^7^, ^8^, ^9^

Pre-clinical studies of therapeutic constructs for DMD are constrained by the approximately 5-kb size limit for a single-stranded AAV vector genome.^10^ Consequently, packaging the entire 13.9-kb cDNA of the muscle-specific isoform of dystrophin into a single AAV capsid cannot be achieved. Although *in vivo* recombination of two or three AAV vector genomes has been demonstrated to generate a mini- or full-length dystrophin-coding sequence,^11^, ^12^ the efficiency of this approach is suboptimal and increases the overall dose of viral capsid proteins. The feasibility of AAV-mediated gene therapy has, however, been demonstrated by the delivery of several micro-dystrophin (μDys) expression cassettes to DMD animal models.

The design of μDys has evolved from two initial observations. First, the dystrophin C-terminal domain was found to be non-essential, due to redundant protein-protein interaction domains within the dystrophin-glycoprotein complex (DGC).^13^, ^14^, ^15^ Second, several very mildly affected Becker muscular dystrophy patients were identified who carried large gene deletions that removed the coding region for approximately 18 of the 24 spectrin-like repeats (SRs) that form the dystrophin central rod domain.^16^, ^17^, ^18^ Early generation μDys lacking the C-terminal domain and up to 20 SRs were highly effective at halting necrosis in dystrophic *mdx* mouse muscles, and they could be encoded on cDNAs less than 3.7 kb in size.^19^, ^20^, ^21^

All miniaturized dystrophins described to date display at least some functional deficiencies; hence, we have been exploring variants of the full-length protein sequence in an attempt to develop μDys with improved function. The full-length muscle isoform of dystrophin plays a mechanical role in transmitting contractile forces laterally through the sarcolemma to the extracellular matrix.^22^ Dystrophin also serves as a scaffold for several signaling proteins.^23^ The amino-terminal domain of dystrophin binds to γ-actin filaments in the subsarcolemmal cytoskeleton.^24^ The central rod domain is the largest portion of dystrophin, and it is composed of 24 SRs that are flanked and interspersed with at least four hinge sub-domains.^16^, ^25^ The rod domain gives dystrophin the necessary elasticity and flexibility for maintaining the integrity of the sarcolemma during muscle contractility.^26^ Various SRs provide unique regions that serve as additional binding sites for cytoskeletal proteins, the sarcolemma, and syntrophin.^27^, ^28^, ^29^, ^30^, ^31^ The cysteine-rich domain and a WW domain in the adjacent hinge 4 region form the β-dystroglycan-binding domain (DgBD), while the carboxy-terminal domain is a scaffold for various isoforms of syntrophin and dystrobrevin.^23^, ^32^, ^33^, ^34^, ^35^, ^36^

Partially functional μDys improve the dystrophic pathology in striated muscles of dystrophic mouse and canine models for DMD by protecting the sarcolemma from contraction-induced injury and increasing force generation.^19^, ^23^, ^37^, ^38^ These parameters are achieved by binding to γ-actin filaments and β-dystroglycan through the amino-terminal domain and the DgBD, respectively, thus providing a mechanically strong link between the subsarcolemmal cytoskeleton and the extracellular matrix.^14^, ^19^, ^26^, ^39^, ^40^ Prior studies indicated these two critical domains must be connected by at least four SRs from the central rod domain, but there are numerous ways in which such miniaturized dystrophins can be constructed. Although several different μDys carrying unique combinations of SRs have been shown to improve the dystrophic pathophysiology, other SR structures have yielded proteins with reduced or minimal functional capacity.^19^ For example, the first μDys we designed, ΔR4-R23/ΔCT (also known as μDysH2) halts muscle necrosis and increases muscle strength, but it was observed to lead to ringbinden in some myofibers subsequent to myotendinous junction injury.^19^, ^41^ Ringbinden was due to a polyproline tract in hinge 2, and it was prevented by the replacement of hinge 2 with hinge 3.^42^ This first-generation ΔR4-R23/ΔCT μDys is currently being tested by Sarepta in a human clinical trial in conjunction with the striated muscle-specific MHCK7 regulatory cassette (RC) that was also developed by our group (ClinicalTrials.gov: NCT03375164).^19^, ^43^

The reasons for the observed functional differences between dystrophin constructs are not always clear, but they are influenced by the presence or absence of specific binding sites for members of the DGC. For example, neuronal nitric oxide synthase (nNOS) is an important signaling protein required for vasodilation in response to muscle contractile activity,^44^, ^45^, ^46^ and the proper association of nNOS with dystrophin requires a syntrophin-binding domain located in SRs 16 and 17.^30^, ^31^ Sequences within SRs 20–24 as well as hinge 4 facilitate the association of dystrophin with microtubules, which contribute to maintaining the intracellular architecture and torque production in skeletal muscle.^47^, ^48^ The juxtaposition of different SRs and hinges that are not adjacent to one another in the full-length protein also affect the tertiary structure, stability, and solubility of μDys.^19^, ^42^, ^49^, ^50^ Nonetheless, the carboxy-terminal and most of the SR domains can be removed from dystrophin with only modest reductions of striated muscle performance.^15^, ^19^ The variable degrees of effectiveness of μDys tested to date suggested that versions with improved function might be designed.^19^, ^31^, ^42^ Indeed, since current systemic delivery methods for AAV vectors have not resulted in complete transduction of all the muscle cells of large animal models, therapeutic interventions would benefit from the use of the most functional μDys available.^38^, ^51^, ^52^, ^53^

In this study, we designed additional μDys with a focus on increasing muscle strength while allowing more complete restoration of the DGC. These designs were directly compared with our previous best μDys, μDysH3, which is highly functional in striated muscles of *mdx* mice and *cxmd* dogs.^42^, ^51^ The design of these constructs focused on the central rod domain in efforts to improve the strength of muscles expressing the constructs and to prevent the ischemia, edema, and fatigue that result from mislocalized nNOS.^31^, ^45^, ^46^, ^54^, ^55^ We also tested the functional capacity and the ability to deliver constructs encoding 4, 5, or 6 SRs. To allow stable packaging of the larger μDys cDNAs, they were ligated to a 436-bp RC modified from the mouse muscle creatine kinase gene basal promoter and upstream enhancer. This CK8e RC displays strong skeletal and cardiac muscle-restricted expression.^2^, ^56^, ^57^ Our findings show that refinements of the μDys structure can generate proteins with enhanced functional properties and stable expression while enabling systemic delivery using AAV vectors. We suggest that these latest constructs have significant potential for gene therapy of DMD.

## Results

### Design of μDys Clones

Most μDys tested to date contained 4 or 5 SR domains, with or without an internal hinge domain, plus the N-terminal actin-binding domain (N-ABD) and the dystroglycan-binding domain (DgBD).^6^, ^15^, ^19^, ^20^, ^31^, ^42^ However, since none of these μDys proteins displayed full functional activity and there are thousands of ways to construct a μDys from the 24 SRs and multiple hinge regions in the full-length protein, we designed seven more proteins to test several unique variations of the rod domain structure. Each of the new clones retained coding sequences for the N-ABD and the DgBD but incorporated novel combinations of SR and hinge domains, with the goal of generating μDys proteins with improved functional properties that could be delivered and expressed from an AAV vector. Since previous studies suggested that some μDys proteins carrying various combinations of hinges and SRs are not functional^19^ and that the degree of functionality is not readily predictable, the latest designs were tested *in viv*o in the *mdx*^4cv^ mouse model for DMD.^58^

**Figure 1:**
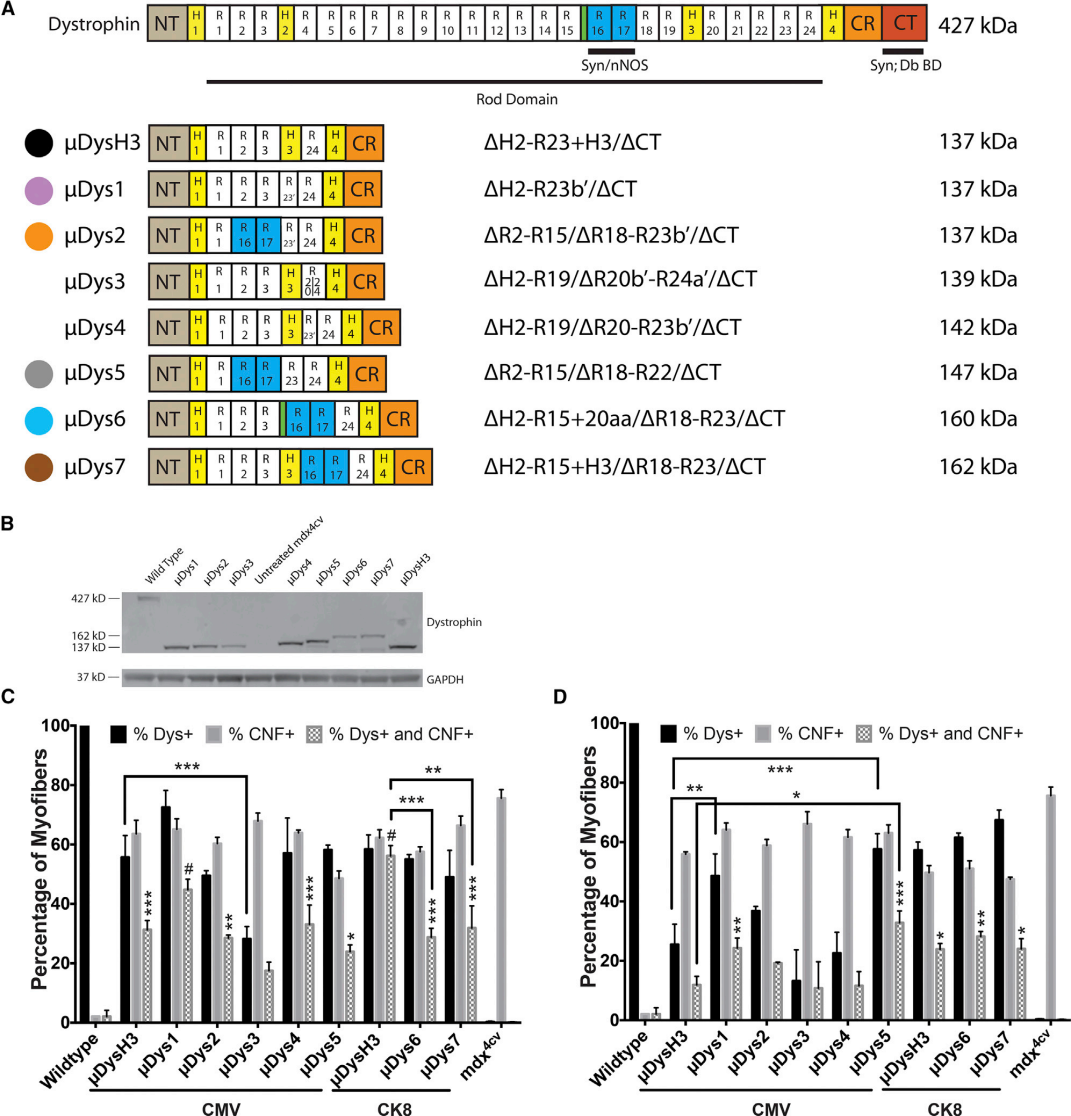
Initial Screening of μDys Designs (A) Schematic of truncated dystrophin constructs used in this study. NT, amino terminal actin-binding domain; H, hinge; R, spectrin-like repeat; nNOS, syntrophin-binding domain that enables localization of neuronal nitric oxide synthase; CR, cysteine-rich domain; CT, carboxyl terminal domain; Syn;Db BD, syntrophin- and dystrobrevin-binding domains. Green unlabeled region marks a 20-amino acid linker between R15 and R16. (B) Dystrophic *mdx*^4cv^ mice were injected with 5 × 10^10^ vector genomes (vg) AAV/CMV-μDys into one tibialis anterior (TA) muscle while the contralateral muscle served as an internal, untreated control. Expression of all tested constructs was verified at 4 weeks after treatment by western analysis of TA muscle lysates, along with wild-type and untreated *mdx*^4cv^ controls. Glyceraldehyde-3-phosphate dehydrogenase (GAPDH) served as an internal loading control. (C and D) Myofibers from TA cross sections were quantified for dystrophin expression and central nucleation at 4 (C) or 12 (D) weeks post-treatment (N = 3–5 per cohort for each time point, mean ± SEM). μDysH3 served as a comparative gauge of performance. μDys6 and μDys7 were too large to be cloned into AAV-expression vectors using the ubiquitous cytomegalovirus (CMV) promoter. Consequently, the CMV promoter was replaced with the striated muscle-specific CK8e promoter to allow efficient packaging and *in vivo* evaluation. This also required μDysH3 to be re-evaluated with this regulatory expression cassette. (C and D) Significance is compared between cohorts (brackets) or with wild-type mice (unbracketed). *p < 0.05, **p < 0.01, ***p < 0.001, #p < 0.0001.

Our previous studies showed that different hinge domains within a μDys can significantly impact its function.^19^, ^42^ Thus, we asked whether alternative and shorter hinges could substitute for hinge 3, which was used in our previous design, μDysH3 (Figure 1A).^42^ Since other studies have also shown that including SRs 16–17 can improve the function of μDys by facilitating nNOS localization to the DGC via α-syntrophin binding,^30^, ^31^we also tested these SRs in the context of various hinge domains and other SRs. We sought to minimize the creation of junctions where domains not normally adjacent to one another in the full-length protein are juxtaposed. Finally, we asked whether the inclusion of some combinations of either 5 or 6 SRs could improve μDys function in the context of the newer hinge and nNOS localization domains tested. The structure of these μDys proteins, in comparison with μDysH3 and the full-length protein, is illustrated in Figure 1.

Two regions in dystrophin were tested for the ability to substitute for hinge 3. The hinge regions of the rod domain are proline rich and lack α-helical signature motifs that compose the triple-helical coiled coil of a SR.^25^ Interestingly, SR23 contains a proline-rich linker between alpha-helices *b* and *c*. We sought to determine if this sequence (together with alpha-helix *c* of SR23) could be used as a hinge domain either by itself (μDys1), adjoining R16-17 (μDys2), or together with H3 (μDys4). An additional construct replaced hinge 3 with the entire SR23 (μDys5; Figure 1A); and, we also tested a second hinge-like region composed of a naturally occurring 20-amino acid structural motif located between SRs 15 and 16 (μDys6).^25^

Additional constructs were designed to test various combinations of the SR domains in the context of these hinges. Previous results have suggested that sequences adjacent to specific SR domains can affect their function,^19^, ^42^ thus we also tested whether a hybrid SR, composed of the N-terminal half of SR20 and the C-terminal half of SR24, would improve function (μDys3). This hybrid SR thus merged the portion of SR20 normally adjacent to hinge 3 with the portion of SR24 that abuts hinge 4 (Figure 1A). Similar considerations influenced the design of the μDys6 construct noted above, where the novel hinge-like region located between SRs 15 and 16 was kept in its normal position adjacent to the syntrophin (and nNOS) localization domain in SR16–17. This latter construct was also compared directly with a similar construct that used hinge 3 instead of the short hinge-like region between SRs 15 and 16 (μDys7). Note that μDys designs 5–7 also incorporated either 5 or 6 SR domains, potentially increasing the overall function of the proteins.^19^, ^20^

### Functionality of Partial Spectrin-like Repeats Is Dependent on the Rod Domain Composition

An initial functional screen of μDys designs 1–7 was made in comparison with the previously characterized μDysH3 by generating AAV6 vectors regulated by the human cytomegalovirus (CMV) immediate early enhancer plus promoter, or in the case of the larger μDys6 and 7, with the CK8e RC. A dose of 5 × 10^10^ vector genomes (vg) was intramuscularly injected into one tibialis anterior (TA) muscle of 5- to 6-week-old dystrophic *mdx*^*4cv*^ male mice,^58^ with the contralateral muscle serving as an internal negative control for expression and morphology. All constructs generated μDys proteins of the predicted sizes, as shown by western analysis (Figure 1B). Dystrophin-positive fibers and central nucleation, a hallmark of necrosis and regeneration, were measured at 4 and 12 weeks post-injection to determine how well each construct was initially expressed, whether expression persisted, and whether the constructs were able to prevent or reduce ongoing myofiber necrosis (Figures 1C and 1D). At 4 weeks post-injection with either RC and at these vector doses, *mdx*^*4cv*^ cohorts treated with any of the μDys designs had 30%–70% μDys-positive fibers. Interestingly, though, the percentage of dystrophin-positive myofibers declined by 12 weeks in TA muscles injected with AAV6 carrying any of the CMV-μDys designs, whereas positive myofibers persisted longer in TA muscles injected with μDys designs expressed via the CK8e RC. These data reflect an advantage of using muscle-restricted RCs over the CMV RC. Treated *mdx*^*4cv*^ muscles also exhibited fewer centrally nucleated fibers (CNFs) than untreated *mdx*^*4cv*^ muscles; and, importantly, μDys-positive myofibers were significantly less likely to be centrally nucleated than μDys-negative myofibers (Figures 1C and 1D).

These assays also disclosed functional (and/or stability) differences among many of the μDys with respect to their effects on both persistence of dystrophin-positive myofibers and CNFs. μDys3 and μDys4 performed notably less well than μDysH3, as evidenced by a reduction in dystrophin-positive myofibers between 4 and 12 weeks post-injection, and μDys1, 2, and 5 exhibited more dystrophin-positive myofibers than μDysH3 by 12 weeks post-injection (Figure 1D). The initial screen of μDys6 and μDys7 used the smaller CK8e RC rather than CMV to enable these larger constructs to be carried by AAV. Both μDys6 and μDys7 generated comparable levels of transduced (Dys+) myofibers and reductions of CNFs by 12 weeks post-injection relative to μDysH3 (Figure 1D). Dystrophin-positive myofibers that were centrally nucleated (Dys+ and CNF+) were also quantified at both time points (Figures 1C and 1D). The proportion of Dys+ myofibers that were centrally nucleated decreased from 4 to 12 weeks post-injection in the treated cohorts, yet remained higher than in wild-type muscles. These results confirm our previous observation that the induction of μDys expression in dystrophic skeletal muscles leads to a slow reduction in central nucleation.^19^

### μDys Constructs Attenuate Pathology in Respiratory and Hind Limb Skeletal Muscles

**Figure 2:**
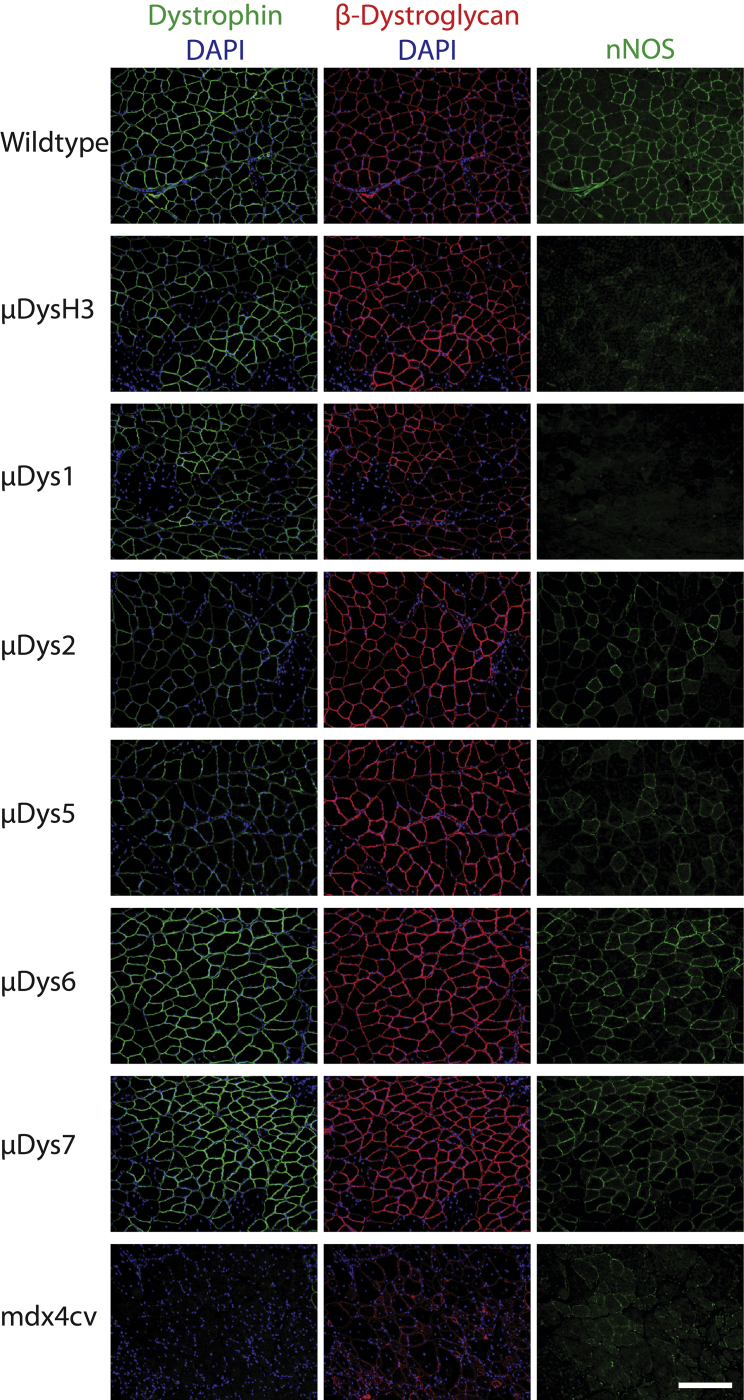
Differential Recruitment of nNOS, but Not β-dystroglycan, by Various μDys Proteins Dystrophic *mdx*^4cv^ mice were injected retro-orbitally with the indicated μDys vectors at 14 days of age. Skeletal muscles were harvested 6 months post-treatment and immunostained for dystrophin-glycoprotein complex members and for nuclei with DAPI. Shown are representative gastrocnemius cryosections. Left column, dystrophin (green) and DAPI (blue); middle, β-dystroglycan (red) and DAPI; right, neuronal nitric oxide synthase (green). Scale bar, 200 μm.

To acquire a more complete assessment of the relative functions of different μDys constructs, we performed further evaluation via a systemic delivery route. For these studies the μDys1, 2, and 5 vectors were re-cloned to replace the ubiquitously active CMV RC with the smaller and muscle-specific CK8e RC, facilitating a direct comparison with the larger, six SR-containing constructs (μDys6 and 7). Due to the sub-optimal performance of μDys3 and 4 noted above, we did not further test those clones. 14-day-old *mdx*^*4cv*^ male mice were infused with vector via retro-orbital sinus injection. Treated mice were assessed at 6 months post-injection, along with age-matched untreated and wild-type controls. This experiment was designed to monitor expression of the μDys constructs and assess the relative extent to which they could halt dystrophic pathophysiology. μDys expression and co-localization with the DGC members β-dystroglycan and nNOS were measured by immunofluorescent staining of gastrocnemius and diaphragm muscle cryosections (Figure 2; Table 1). This analysis indicated that the μDys designs restored β-dystroglycan localization in essentially all μDys-positive fibers. In contrast, restoration of nNOS localization occurred only with μDys2, μDys5, μDys6, and μDys7 treatment; and, as anticipated, no nNOS restoration was seen with μDysH3 or μDys1 treatment, since these designs lack the syntrophin (and nNOS) localization domain in SR16–17 (Figure 1A).

**Table 1:** Histology and Specific Force of Systemically Treated *mdx*^4cv^ Mice at 6 Months Post-treatment

Group	Percentage of Dystrophin-Positive Fibers (Dys^+^)		Percentage of Centrally Nucleated Fibers (CNF^+^)		Percentage of Dys^+^ and CNF^+^ Fibers		Specific Force (kN/m^2^)	
	Gastrocnemius	Diaphragm	Gastrocnemius	Diaphragm	Gastrocnemius	Diaphragm	Gastrocnemius	Diaphragm
Wild-type	100***	100***	2 ± 1***	2 ± 1***	2 ± 1	2 ± 1	200 ± 16**	130 ± 13*
μDysH3	64 ± 5##,***	85 ± 4***	8 ± 2***	3 ± 1***	< 1	2 ± 1	201 ± 7**	89 ± 12
μDys1	65 ± 3##,***	80 ± 6***	12 ± 2***	3 ± 1***	2 ± 1	< 1	187 ± 11	103 ± 7
μDys2	31 ± 10###,**	53 ± 9###,***	43 ± 5###,*	14 ± 4##, ***	3 ± 1	1 ± 1	210 ± 8**	127 ± 26
μDys5	70 ± 4#,***	74 ± 9***	14 ± 3***	7 ± 4***	1 ± 1	1 ± 1	225 ± 11***	148 ± 27*
μDys6	60 ± 4##,***	78 ± 5***	17 ± 4***	2 ± 1***	3 ± 1	< 1	199 ± 9*	81 ± 3
μDys7	84 ± 5***	91 ± 2***	19 ± 3#,***	10 ± 1***	9 ± 1	8 ± 1	193 ± 8**	99 ± 14
*mdx*^*4cv*^	3 ± 1###	2 ± 1###	58 ± 1###	32 ± 1###	1 ± 1	< 1	132 ± 7##	57 ± 5#

Values represent means ± SEM. #p < 0.05, ##p < 0.01, ###p < 0.001, statistically significant difference from wild-type line. *p < 0.05, **p < 0.01, ***p < 0.001, statistically significant difference from *mdx*^4CV^. N = 4–9 per group.

**Figure 3:**
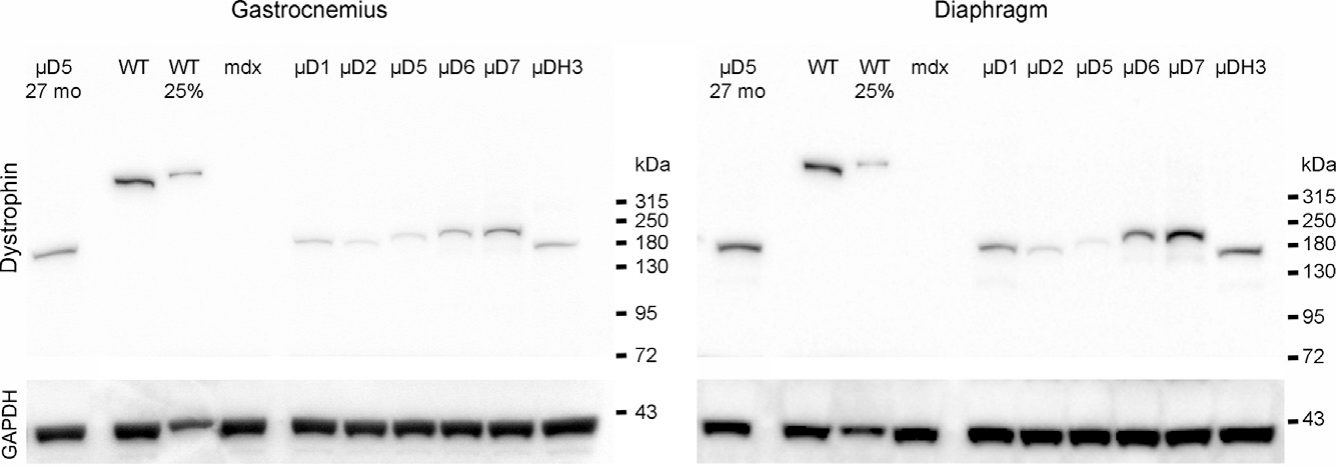
Western Analysis of Micro-dystrophin Expression To monitor the size of μDys protein expression following infusion of the various AAV vectors, 40 μg muscle protein extracted from select muscles was analyzed by western blot at 6 months post-infusion. A sample from a mouse infused 27 months prior to sacrifice is shown on the left of each panel (μD5 27 mo). As a loading control, the lower portion of each blot was cut off and analyzed for GAPDH expression. All μDys proteins migrated at the predicted molecular size. Note that the 27-month-old mice were infused with a different batch of vector and at different times than were the mice analyzed at 6 months post-infusion. WT and WT 25% denote 40 and 10 μg, respectively, protein extracted from wild-type mouse muscles. μD5 etc. refer to muscle extracts from mice infused with AAV/CK8-μDys5 or other μDys design variants.

By 6 months post-treatment, the percentage of dystrophin-positive myofibers that displayed central nucleation was not significantly different from wild-type controls in most groups (Table 1). The μDys7 construct had 8%–9% centrally nucleated myofibers, but this did not rise to a level of significance. Nonetheless, the result suggests a possible functional deficiency for this μDys design. The μDys1-, 5-, 6-, 7-, and H3-injected mice displayed ≥60% dystrophin-positive myofibers in the gastrocnemius and ≥74% in the diaphragm at 6 months. Transduction levels of μDys2 were only 31% in the gastrocnemius muscle and 53% in the diaphragm, making its performance in this assay the worst of the constructs tested; Table 1). Western analysis of gastrocnemius and diaphragm muscle lysates confirmed the expression of μDys proteins corresponding to the predicted sizes of the six different constructs, with significant expression still being observed at 27 months, as demonstrated for μDys5 (Figure 3). While expression appeared higher at 27 months in the samples assayed, we note that different batches of vector were used for the 6 and 27 month analyses (see below).

**Figure 4:**
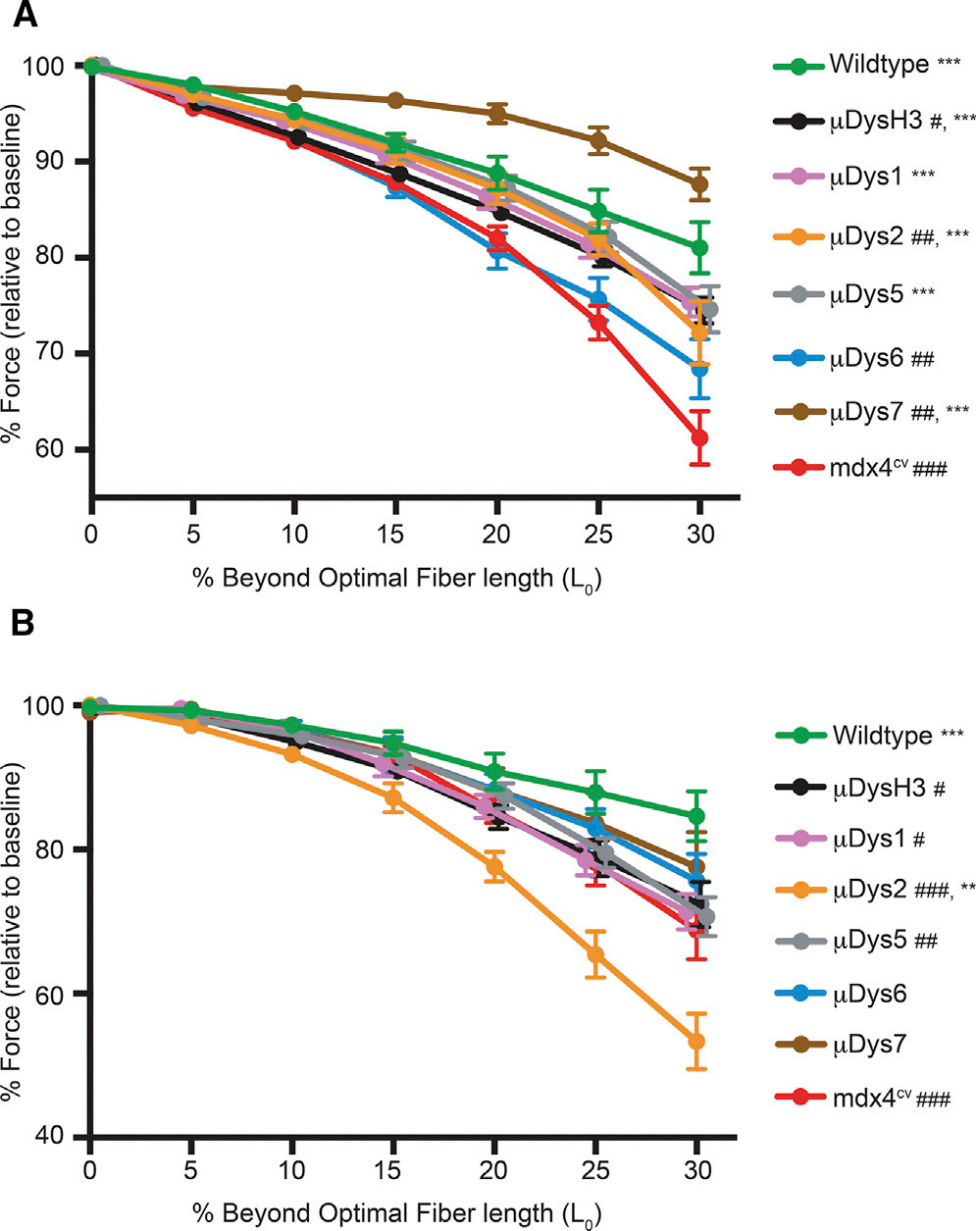
Protection of Muscles from Eccentric Contraction-Induced Injury Muscles from systemically treated mice were subjected to eccentric contractions of increasing length. Gastrocnemius muscles (A) and diaphragm muscle strips (B) were measured for the maximum isometric force generated prior to an eccentric contraction. During stimulating contractions, muscles were lengthened at a defined distance beyond their optimum fiber lengths. Distances are reported as percentage beyond optimal fiber length (L_O_). *p < 0.05, **p < 0.01, ***p < 0.001 from *mdx*^4cv^ at 30% beyond L_O_. #p < 0.05, ##p < 0.01, ###p < 0.001 from wild-type at 30% beyond L_O_.

The specific force-generating capacity of select muscles in all the treated cohorts remained significantly higher than in untreated *mdx*^*4cv*^ muscles at 6 months post-treatment (Table 1). Injection of one construct, μDys5, led to specific force-generating levels not significantly different from those in wild-type mice in both the gastrocnemius and the diaphragm muscles (Table 1). Based on previous studies with mini-dystrophins containing eight SRs, we had predicted that the μDys constructs containing greater numbers of SRs would generate more specific force and provide greater protection from contraction-induced injury than smaller constructs.^19^ While specific force generation in the gastrocnemius muscles of μDys6- and μDys7-treated mice was also significantly higher than in untreated controls (p < 0.05 and p < 0.01, respectively), they were not the highest of the treated cohorts (Table 1). Instead, μDys5-injected mice displayed the highest specific force generation. The larger constructs were also not necessarily the best at protecting from eccentric contraction-induced injury. For example, gastrocnemius muscles from μDys6-injected mice had the largest force deficit, while μDys7-expressing gastrocnemius muscles displayed the greatest protection from contraction-induced injury (Figure 4A). In contrast, resistance to contraction-induced injury in diaphragm muscle strips was fairly similar among all constructs tested, with the exception of μDys2 (Figure 4B). The contrasting results of μDys6 and μDys7 between muscle groups reinforces previous observations that the performance of a particular μDys construct can vary between different types of skeletal muscles.^19^, ^43^ This point was also exemplified with μDys2 treatment, where the susceptibility to contraction-induced injury was reduced in the gastrocnemius but exacerbated in the diaphragm (Figure 4).

### μDys Lacking Hinge 2 Do Not Induce Ringed Myofibers

**Figure 5:**
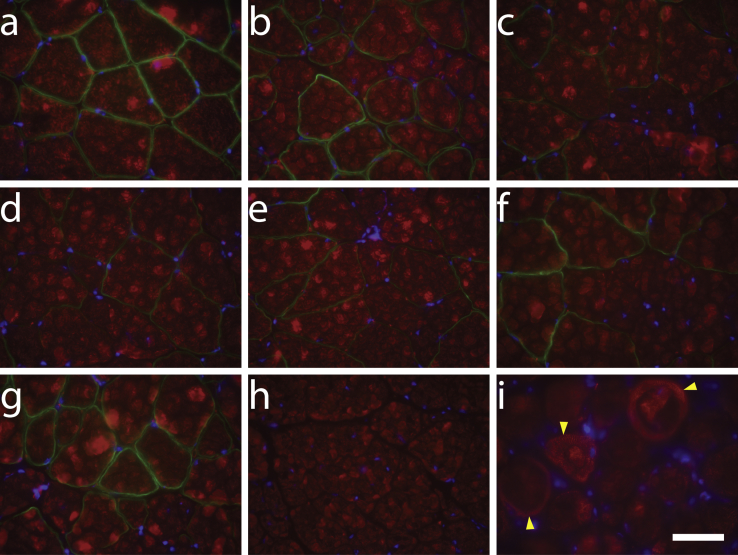
Improved μDys Constructs Do Not Induce Ringbinden in Skeletal Muscles Dystrophic *mdx*^4cv^ mice were injected retro-orbitally with various CK8e-μDys vectors at 14 days of age. At 6 months post-treatment, cryosections of gastrocnemius muscles were immunostained for dystrophin (green) and α-sarcomeric actin (red) and counterstained with DAPI (blue). One representative section is shown from cohorts of wild-type (a); *mdx*^4cv^ treated with μDysH3 (b), μDys1 (c), μDys2 (d), μDys5 (e), μDys6 (f), and μDys7 (g); and untreated *mdx*^4cv^ (h) mice. Gastrocnemius muscle cryosections from transgenic mice expressing ΔR4-R23/ΔCT^19^ on the *mdx*^4cv^ background (i) were also immunostained with α-sarcomeric actin (red) and counterstained with DAPI (blue) as a positive control. Yellow arrowheads mark examples of ringbinden formation around myofibers. Ringed myofibers were only observed with the ΔR4-R23/ΔCT micro-dystrophin.^19^, ^42^ Scale bar, 50 μm.

As noted above, one of our original μDys constructs (μDysH2)^19^ had been shown to be highly functional using several physiological assays but induced ringbinden and neuromuscular junction abnormalities subsequent to myotendinous junction injury in a subset of limb muscles).^41^, ^42^ These abnormal structural properties were shown to be caused by the juxtaposition of a proline-rich tract in hinge 2 with the dystroglycan-binding domain, and they were not observed with similar constructs lacking this polyproline tract (e.g., μDysH3).^42^ To explore whether any of the latest μDys proteins led to ringbinden, we stained gastrocnemius muscles from a set of systemically injected mice for the presence of ringed myofibers (i.e., those containing transversely oriented sarcomeres). As shown in Figure 5, ringbinden was only observed with the ΔR4-R23/ΔCT μDys (μDysH2).

### Persistence of μDys Expression

**Figure 6:**
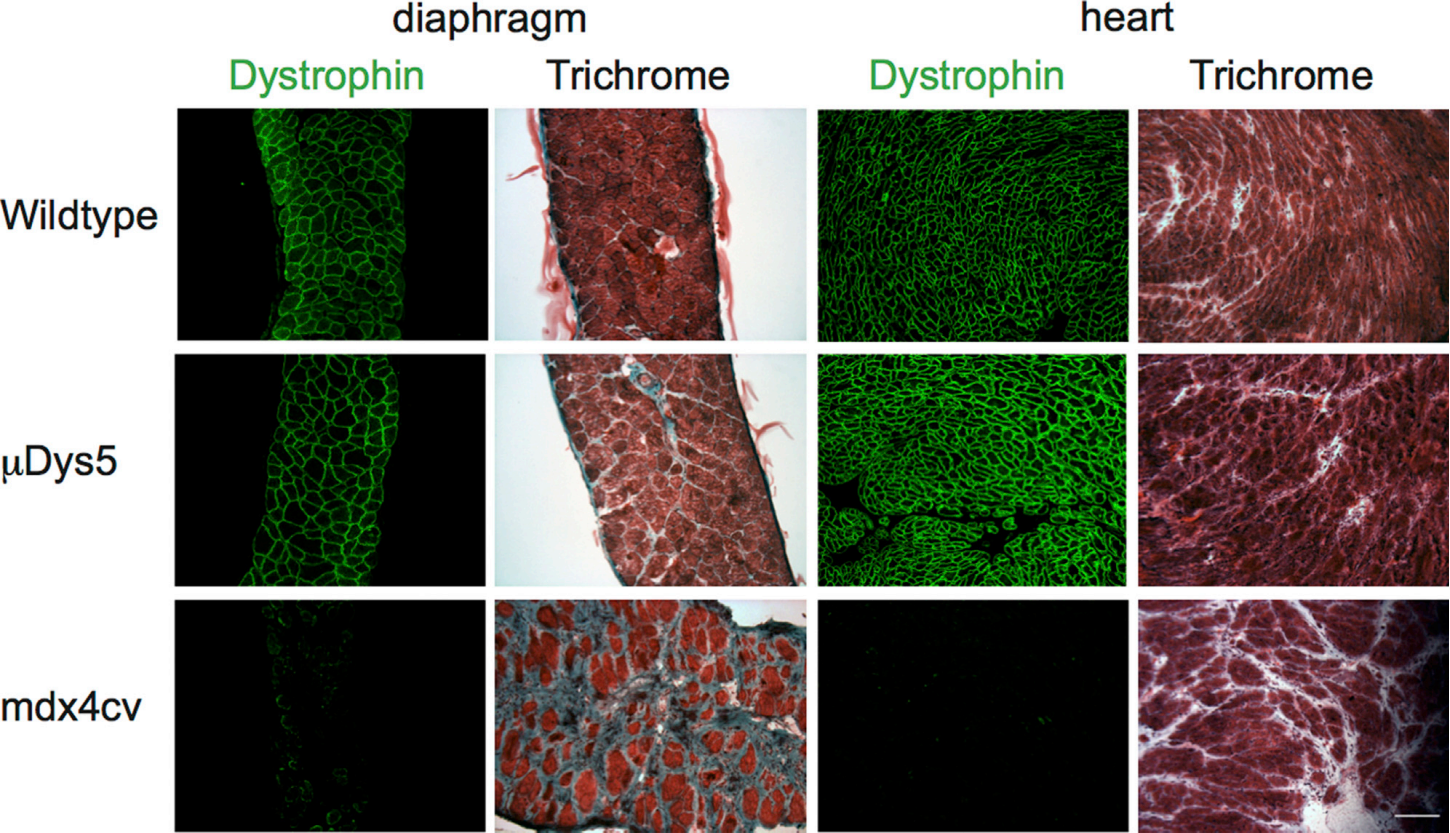
Long-Term Expression of Micro-dystrophin in Dystrophic Mice 2-week-old *mdx*^4cv^ mice were systemically infused with 1 × 10^13^ vg AAV/CK8e-μDys5 or saline and analyzed 27 months later. Wild-type C57BL/6 mice were analyzed at 24 months of age. Muscles were harvested, snap frozen in OCT, and used to prepare cryosections. These sections were either immunostained for dystrophin (green) and counterstained with DAPI (blue) or histochemically stained with H&E and Gomori’s trichrome (Trichrome). Scale bar, 100 μm.

Since μDys5 displayed the best combination of expression levels, force generation, resistance to contraction-induced injury, and vector titer (see the Materials and Methods) among the μDys described in this study, we performed a long-term analysis of *mdx*^4cv^ mice systemically injected with AAV6/CK8e-μDys5. Treated mice were sacrificed 27 months post-vector infusion, and diaphragm and heart muscles were analyzed for histopathology and dystrophin immunostaining. We also analyzed the gastrocnemius and diaphragm muscles from one injected animal by western blot. As shown in Figures 3 and 6, μDys5 was still highly expressed after 27 months (with nearly 100% dystrophin-positive myofibers and myocytes), and the combination of μDys5 structure with its continued transcription via the CK8e RC maintained normal muscle histology in both diaphragm and heart muscles.

## Discussion

Numerous studies in dystrophic animal models reveal that AAV-mediated delivery of μDys constructs is a promising approach for treating DMD.^6^ Despite these encouraging results, it is clear that μDys proteins, whose cDNAs are small enough to be packaged together with muscle-specific RCs in AAV vectors, display functional limitations. These have included, to various degrees, an inability to assemble the entire DGC or support normal muscle mechanical and signaling properties. Transgenic animal studies indicate a positive correlation between the size of the rod domain and the overall functionality of dystrophin, such that dystrophins carrying 8 or more SRs can support normal muscle function.^19^ Although larger dystrophins have been produced in dystrophic mouse muscles by the co-delivery of multiple AAV vectors, the higher vector doses needed to co-deliver two or more vectors to the vast majority of muscle cells combined with low efficiency and the generation of undesired recombinant vector genomes have limited widespread development of this approach.^11^, ^12^ Here we sought to design and test μDys proteins with potentially increased function that could be delivered by a single vector.

Our results demonstrate that altered rod domain composition can lead to μDys proteins with improved function in the *mdx*^4cv^ mouse model of DMD. We have not observed a strict correlation between μDys construct size and overall function, but some generalities can be inferred. For example, in earlier studies, various μDys carrying only 4 SRs displayed significant differences in function, and these differences varied between different skeletal muscle types.^19^, ^21^ Two constructs carrying 6 SR domains performed reasonably well, but their larger size prevented high-titer AAV vector production (Figure 1; Materials and Methods; see also Wang et al.^20^). In contrast, our μDys5 design, which carries 5 SR domains, was the most functional of any μDys tested to date. We note that Wang et al.^20^ previously described two μDys carrying different sets of five SR domains. While those constructs appeared to function well, and one is being tested in a clinical trial (ClinicalTrials.gov: NCT03362502), they have not been directly compared with other μDys and are unable to localize nNOS to the DGC.^31^

Along with increased dystrophin-positive myofibers, improvements in the specific force-generating capacity and protection from eccentric contraction-induced injury were observed at 6 months post-treatment in muscles expressing several of the constructs described here. As noted, μDys5 led to the highest measured specific force in both the gastrocnemius and diaphragm muscles of treated *mdx*^*4cv*^ mice, and its continued high-level expression was maintained for at least 27 months in both skeletal and cardiac muscles. Also encouraging were the results with our largest tested construct containing six SRs, μDys7. At 6 months post-treatment, μDys7-expressing muscles displayed the highest resistance to contraction-induced injury in the gastrocnemius muscle. Despite also having six SRs, μDys6 failed to perform as well as μDys7 in the gastrocnemius 6 months after treatment. The only difference between these two constructs was the middle of the rod domain, suggesting that the 20-amino acid region between SRs 15 and 16 does not function effectively as a surrogate hinge.

Four of the μDys constructs incorporated the α-syntrophin-binding domain in SR16–17, which is required to localize nNOS to the DGC.^30^, ^31^ Since sarcolemmal localization of nNOS prevents exercise-induced fatigue, ischemia, and edema and reduces overall dystrophic pathophysiology,^45^, ^46^, ^55^ μDys 2, 5, 6, and 7 (Figure 2) would thus be predicted to counteract these deficits. Additionally, we assessed whether expression of any of these constructs caused ringbinden formation in myofibers, since this pathological phenotype was observed in some muscles of transgenic or vector-treated mice expressing the ΔR4-R23/ΔCT (μDysH2) construct.^19^, ^41^, ^42^ With the exception of μDysH2, no ringbinden formation was observed following the expression of any of the newer constructs in the gastrocnemius or any other skeletal muscles in systemically treated *mdx*^4cv^ mice (Figure 5; data not shown).^42^ We note that the ringbinden-inducing μDysH2 (which also lacks the nNOS localization domain) is being tested in a human clinical trial (ClinicalTrials.gov: NCT03375164).

Generating transgenic mice expressing all the constructs tested in this work would perhaps allow a more stringent characterization of these modified rod domain compositions. However, our systemic delivery study is arguably more translatable, since it also determined how effective our standard and improved constructs were at halting the dystrophic pathogenesis in post-natal animals. This approach also factors in the doses administered and the time of therapeutic intervention. Our study further incorporated the variable expression levels among myofiber types when using the myogenic-specific CK8e RC. Previous myogenic-specific RCs exhibited higher expression in fast, type II myofibers, as identified by the expression of myosin heavy-chain isoforms.^59^ The CK8e RC used in this study also displays similar differences in expression among myofiber types (S.D.H., unpublished data); but, as shown in Figures 3 and 6, the CK8e RC provided robust long-term expression of μDys5 in both skeletal and cardiac muscles.

Our studies have identified two designs, μDys5 and μDys7, that function better than our previously best μDys, the μDysH3 construct.^42^ However, no single μDys construct dominated in every test used or at all time points measured. This could be attributed to the experimental variability between animals (transcription and translation rates for individual sequences) and the consideration that all treatments of dystrophic mice were either after or immediately prior to the onset of myofiber necrosis and regeneration. We recently reported that a canine analog of μDys5 performed well in the *mdx*/Dba2 mouse model for DMD, although that study did not compare μDys5 with other constructs.^60^ The *mdx*/Dba2 model was a potential platform to test heart function, but the identification of a cardiac phenotype in the parental Dba/2 strain complicated the assessment of cardiac function.^60^ Previous studies of μDys in the hearts of *mdx* mice have suggested an incomplete correction of systolic function,^61^, ^62^, ^63^ but overall assessment has been limited by a lack of robust studies in animal models that progress to heart failure. Consequently, further studies will be needed to determine if one construct small enough to be packaged within a single AAV vector genome will suffice for all muscle groups. Additionally, regulatory expression cassettes can also be refined for improved expression in certain anatomical muscles, such as heart and diaphragm, and potentially for fiber types within these (S.D.H., unpublished data). Nonetheless, the Ck8e-μDys5 combination appears highly promising, and it is currently being tested in a DMD clinical trial by Solid Biosciences (ClinicalTrials.gov: NCT03368742).

## Materials and Methods

### Animal Experiments

Male wild-type (C57BL/6) and dystrophic *mdx*^*4cv*^ mice were used in this study. Animal experiments were performed in accordance with and approved by the Institutional Animal Care and Use Committee of the University of Washington. All experiments conformed to all relevant regulatory standards. For initial screening, dystrophic *mdx*^*4cv*^ mice (5–6 weeks old) were administered 5 × 10^10^ vg recombinant AAV6 vector into the TA muscle (N = 3–5). Control mice were injected with Hank’s balanced saline solution as a sham manipulation. In systemic administration studies, 14-day-old *mdx*^*4cv*^ males were administered 1 × 10^13^ vg recombinant AAV6 vector via retro-orbital sinus injection (N = 4–9). Animal numbers were chosen based on previous studies. Male *mdx*^*4cv*^ pups were chosen at random and based on availability in our breeding colony. To blind the study and avoid potential bias, mice were given an identification number at necropsy that differed from their colony number. Necropsy numbers were assigned by researchers who didn’t conduct AAV vector injections. The identification and treatment history of each mouse was determined after analysis.

### Vector Cloning and Virus Production

All μDys transgenes were engineered using standard cloning techniques. Modified regions were subcloned starting with μDysH3 (ΔH2-R23/ΔCT,+H3), using MfeI/XhoI or NheI/XhoI restriction sites flanking the majority of the central rod domain.^42^ A polyadenylation signal engineered from the rabbit beta-globin gene was subcloned immediately 3′ of the μDys cDNA.^42^ RCs to drive gene expression of μDys cDNAs were derived from either the human CMV immediate early enhancer plus promoter (CMV) or modified enhancer and promoter sequences from the mouse muscle creatine kinase gene (CK8e). Recombinant AAV6 vectors were prepared in 850-cm^2^ roller bottles (RBs) as previously described.^9^, ^64^ Purified vectors were quantified by Southern analysis and qPCR (vg/mL). To ensure approximately equal dosing in treating dystrophic mice, all vector preparations used in this study were titered in parallel, as individual titers are difficult to quantify precisely. Average yields varied between a low of 1.3 × 10^12^ vg/RB (μDys6 and 7) to a high of 4.5 × 10^12^ vg/RB (μDys5).

### Immunoblotting

TA, gastrocnemius, and diaphragm muscles of mice were snap frozen in liquid nitrogen and then ground by dry ice-chilled mortar and pestle under liquid nitrogen. Muscles were homogenized in lysis buffer (1% Triton X-100, 50 mM Tris-HCl [pH 7.5], 150 mM NaCl, and 1 mM EDTA) for the initial screen of TA muscles and radioimmunoprecipitation (RIPA) buffer containing 5 mM EDTA for gastrocnemius and diaphragm muscles; both lysis buffers were further supplemented with complete mini protease inhibitor cocktail at the manufacturer’s recommended concentration (Roche, Indianapolis, IN). Protein concentrations of the lysates were determined using the Coomassie Plus Bradford Assay (TA) or BCA assay (gastrocnemius and diaphragm) (Pierce, Rockford, IL). 40 μg protein was suspended in NuPage (TA) or Bolt (gastrocnemius and diaphragm) LDS sample buffer (Life Technologies) supplemented with 100 mM dithiothreitol and loaded onto a NuPAGE/Bolt 4%–12% Bis-Tris polyacrylamide gel (Life Technologies). After running the gels and transferring samples onto Amersham Hybond-P polyvinylidene fluoride membrane (GE Healthcare Life Sciences, Piscataway, NJ), blots were blocked with 10% (TA) or 5% (gastrocnemius and diaphragm) nonfat dry milk in PBS (TA) or Tris-buffered saline (TBS) (gastrocnemius and diaphragm). Blots were then incubated 1 h with primary antibodies in 5% nonfat dry milk and 0.1% Tween-20 in PBS or TBS. After washing three times in PBST or TBST, membranes were incubated 1 h in secondary antibodies plus 5% nonfat milk in PBST or TBST, followed by four washes in PBST or TBST. Primary antibodies included mouse anti-dystrophin (MANEX1011B clone 1C7, Developmental Studies Hybridoma Bank [DSHB]) and rabbit anti-glyceraldehyde 3-phosphate dehydrogenase (G9545, Sigma) as a loading control. Secondary antibodies included horseradish peroxidase-conjugated donkey anti-rabbit or anti-mouse (Jackson ImmunoResearch Laboratories, West Grove, PA). Blots were developed with Pierce ECL Plus western blotting substrate (Thermo Fisher Scientific) or Clarity western ECL substrate (Bio-Rad) and scanned using a Storm 860 imaging system (GE Life Sciences) or a Chemidoc MP imaging system (Bio-Rad).

### Functional Analyses of Skeletal Muscles

Muscles were assayed *in situ* (gastrocnemius) and *in vitro* (diaphragm) for specific force generation and susceptibility to contraction-induced injury, as previously described with the noted modifications.^42^, ^63^, ^65^ The maximum isometric force was determined at optimal muscle fiber length, and then the muscle was subjected to a series of progressively increasing length changes under stimulation (model 701C, Aurora Scientific). Maximum isometric tetanic force was measured by stimulating at 150 and 180 Hz for the gastrocnemius and diaphragm, respectively. Eccentric contractions were performed by first stimulating at a fixed length, allowing peak isometric force to be generated, for 150 ms (gastrocnemius) or 100 ms (diaphragm). Immediately following this initial period, the muscle remained stimulated for an additional 200 ms (gastrocnemius) or 300 ms (diaphragm) while the muscle was physically lengthened to the desired length. Once stretched to the desired length, stimulation ceased and the muscle was returned to resting length. The muscle rested for 30 s before the subsequent eccentric contraction. A series of length changes, or strains, of 0%–30% of the optimum length was applied to potentiate overloading of the contractile properties and damage to the muscle architecture. The response from an eccentric contraction was measured by the peak isometric force generated just prior to the subsequent eccentric contraction.

Mice were anesthetized with 2,2,2-tribromethanol (Sigma) to be unresponsive to tactile stimuli and then prepped for *in situ* analysis of the gastrocnemius. The Achilles tendon was exposed by incision at the ankle, sutured with 3-0 braided silk (Ethicon, Cincinnati, OH), severed, and secured to the lever arm of a dual-mode force transducer-servomotor (model 305B-LR, Aurora Scientific, Aurora, ON, Canada). Mice were immobilized and secured to the apparatus by a stainless steel pin inserted through the knee and by taping the hind paw to a customized Plexiglas platform. Gastrocnemius muscle was stimulated via two needle electrodes that were inserted through the skin on either side of the peroneal nerve in the region between the knee and hip. The servomotor’s position was manipulated on three axes to help determine the optimal muscle fiber length. The servomotor was controlled by LabView software that also allowed data acquisition (National Instruments, Austin, TX).

For *in vitro* preparations of diaphragm, the anesthetized mouse was sacrificed after gastrocnemius analysis and the entire diaphragm muscle and surrounding ribcage was quickly excised to a dish containing oxygenated Tyrode’s solution (bubbled with 5% CO_2_-95% O_2_ [pH 7.3]) containing the following: 121 mM NaCl, 5 mM KCl, 1.8 mM CaCl_2_, 0.5 mM MgCl_2_, 0.4 mM NaH_2_PO_4_, 24 mM NaHCO_3_, and 5.5 mM glucose. A diaphragm strip composed of longitudinally arranged full-length muscle fibers, a portion of the central tendon, and a portion of rib bones and intercostal muscle on the distal end of the strip was isolated under a microscope. The muscle strip was tied with needle-lead braided surgical silk (6-0, P1; Ethicon) at the central tendon, sutured through the rib bone portion (5-0; Ethicon), and then secured to an *in situ* mouse apparatus with a temperature-controlled, horizontal bath (model 809A, Aurora Scientific). The apparatus bath was filled with bubbled Tyrode’s solution (described above) and maintained at 25°C.

Optimal fiber length was determined, and isometric and eccentric contractile properties were assessed in a manner similar to gastrocnemius muscle analysis, with the conditions specified above for the diaphragm muscle. The specific force of both muscle groups was determined by normalizing maximum isometric force to the mass of the gastrocnemius muscle or diaphragm strip, respectively. The following equation was used: specific force = maximum force × pennation × muscle length × 1.04 density/muscle weight.^66^ (Pennation is the angle at which bundles of skeletal muscle fibers orient themselves between the tendons of the muscle. For the gastrocnemius muscle, this angle was determined by a previous study.^42^) Diaphragm muscle strips were isolated in such a way that the myofibers would contract directly between the semitendinosus junction and the myotendinous junction at the rib.^67^ Pennation for the gastrocnemius and diaphragm equaled 0.45 and 1, respectively.

### Histological Analysis

After physiological analysis, mice were sacrificed for necropsy. Muscles were embedded in Tissue-Tek OCT medium (Sakura Finetek USA, Torrance, CA) and frozen in liquid nitrogen-cooled isopentane. Transverse sections approximately 10 μm thick were used for immunofluorescence and histological studies. For immunostaining, sections were blocked in 2% normal goat serum and 1% Tween-20 in potassium PBS (KPBS). Sections were washed with 0.2% gelatin in potassium PBS (KPBS-G), followed by an incubation in primary antibodies diluted in 2% normal goat serum in KPBS-G. Sections were then rinsed in KPBS-G three times before incubation with secondary antibodies and DAPI (Sigma, St. Louis MO). After washing three more times in KPBS-G, slides were mounted in ProLong Gold antifade reagent (Life Technologies, Grand Island, NY).

Primary antibodies included rabbit polyclonal N-terminal anti-dystrophin antibody,^14^ mouse monoclonal anti-dystrophin (MANEX1011B clone 1C7, DSHB at the University of Iowa, Iowa City, IA) conjugated to Alexa 488 (Life Technologies), mouse anti-β-dystroglycan (MANDAG2 clone 7D11, DSHB) conjugated to DyLight-594 (Thermo Fisher Scientific, Rockford, IL), rat anti-α2-laminin (clone 4H8-2, Sigma, St. Louis, MO), and rabbit anti-nNOS (Z-RNN3, Life Technologies). Secondary antibodies were goat anti-rabbit or anti-rat conjugated to Alexa-660 or Alexa-594, respectively (Life Technologies). Images were captured on an Olympus SZX16 dissection fluorescent microscope with DP software (Oympus, Center Valley, PA). All myofibers in transverse sections from near the mid-belly of the muscle were scored for dystrophin immunoreactivity. For histological assays, cryosections were stained with H&E and/or Gomori’s trichrome (Thermo Fisher Scientific, Waltham, MA).

### Statistical Analysis

All results are reported as mean ± SEM. Differences between cohorts were determined using one-way and two-way ANOVA with Tukey’s post hoc multiple comparison test. All data analyses were performed with GraphPad Prism 6 software (San Diego, CA).

## Author Contributions

Conceptualization, J.S.C. and S.D.H; Methodology, J.N.R., K.H., N.E.B., J.M.A., and J.S.C.; Investigation, J.N.R., K.H., N.E.B., and J.M.A.; Formal Analysis, J.N.R., K.H., N.E.B., S.D.H., and J.S.C.; Writing – Original Draft, J.N.R.; Writing – Review and Editing, S.D.H. and J.S.C.; Visualization, J.N.R. and K.H.; Supervision, J.M.A., S.D.H., and J.S.C.

## Conflicts of Interest

J.N.R., J.S.C., and S.D.H. are the sole inventors on patents covering the various μDys and MCK RCs shown in Figure 1. J.S.C. is an inventor on patents covering the ΔR4-R23/ΔCT μDys (μDysH2). J.S.C. holds equity in, and is a member of the scientific advisory board of, Solid Biosciences.
